# Epigenetic Mechanisms in Obesity: Broadening Our Understanding of the Disease

**DOI:** 10.7759/cureus.47875

**Published:** 2023-10-28

**Authors:** Erin N. L. Smith, Merin Chandanathil, Richard M Millis

**Affiliations:** 1 Graduate Studies, American University of Antigua, St. Johns, ATG; 2 Pathophysiology, American University of Antigua, St. Johns, ATG

**Keywords:** arb3, npy, pomc, t3, gestational period, high-fat diet, long non-coding rna, micro-rna, histone acetylation, dna methylation

## Abstract

Now recognized as more than just the result of overeating or the consumption of poor-quality foods, obesity is understood to be a multifactorial disease, strongly correlated with a variety of environment-gene interactions. In addressing the complex public health issue of obesity, medical practitioners, along with their allied healthcare counterparts, face the challenge of reducing its prevalence by utilizing and sharing with patients the current, yet incomplete, scientific knowledge concerning the disease. While continued research is required to strengthen direct cause-effect relationships, substantial evidence links post-translational modifications such as DNA methylation and histone modifications of several candidate “obesity” genes to the predilection for obesity. Additional evidence supports the influence of maternal diet during the gestational period, individual diet, and other lifestyle and genetic factors in obesity. The purpose of this review is to synthesize the current information concerning epigenetic modifications that appear to support, or result from, the development of obesity. Such mechanisms may serve as therapeutic targets for developing novel prevention and/or treatment strategies for obesity or as epigenetic biomarkers for monitoring recovery.

## Introduction and background

Since 1975, the global obesity rate has at least tripled [1]. According to 1995 statistics, approximately 200 million adults and 18 million children under the age of five were categorized as being overweight or obese [2]. By 2016, approximately 650 million adults and 39 million children under the age of five were either overweight or obese [1]. Contrary to popular belief, this public health crisis is affecting not only first-world nations but virtually all developing nations. An estimated 115 million individuals in developing nations are afflicted with obesity and the consequences that accompany the disease [2]. Screening for obesity involves measuring the body weight of the patient in kilograms and then dividing that number by the square height of the patient measured in meters. The result of this calculation is the Body Mass Index (BMI). Generally, a patient’s BMI is classified as healthy when it falls within the range of 18.5 to 24 kg/m2. A BMI between 25 and 29 kg/m2 is considered overweight, and a BMI > 30 kg/m2 is considered obese. While BMI has been shown to be a poor direct measure of total body fat, there is still a positive correlation between a high BMI, excess body fat, and unfavorable health outcomes associated with increased body fat [3].

According to 2019 statistics provided by OurWorldData, cardiovascular diseases are the leading cause of death worldwide [4]. High-risk factors for cardiovascular diseases include high blood pressure, diabetes, high cholesterol, physical inactivity, and an unhealthy diet [5]. Notably, there is a very high correlation between the above-mentioned risk factors and obesity [6]. As the connection between obesity and cardiovascular disease continues to be supported by medical evidence, it stands to reason that understanding the causes of obesity should be a high priority for the global healthcare community. Commonly reported causes of obesity include long-term imbalance between energy intake versus energy expenditure, excessive alcohol intake, limited physical activity, and medical conditions such as hypothyroidism, polycystic ovarian syndrome, and Cushing’s syndrome. Other risk factors for obesity that have gained increasing traction include poor sleep quality, poor stress management and emotional instability [7]. Less commonly discussed are the genetic factors that contribute to obesity and the interplay between those factors and the external environment, which is the main focus of epigenetics.

Epigenetic research studies how environmental and lifestyle factors such as climate, diet, water intake, etc. affect the expression of a person's genes and production of their structural and functional proteins. An important difference to note between epigenetic changes to DNA and genetic mutations is that epigenetic changes are reversible and do not affect the base genetic sequence. Although the core DNA remains fundamentally unchanged, epigenetic modifications can positively or negatively influence the way the cell machinery reads DNA; thereby, speeding, slowing or even stopping the production of proteins that are essential for daily functions and energy metabolism [8]. Our current knowledge of epigenetic processes suggests that understanding the epigenetic mechanisms playing a role in obesity may help broaden our understanding of the disease and provide insight into its treatment and prevention. In this review, we highlight some of the principal epigenetic processes involved in obesity and postulate about their clinical relevance.

## Review

DNA methylation

DNA methylation is a type of post-translational modification mediated by a family of enzymes known as DNA methyltransferases (DNMTs). During this process, methyl groups are added, through covalent bonding, to cytosine residues in areas of DNA where cytosine residues are followed by guanine residues (CpG sites) [9]. The result of DNA methylation is gene silencing. This is facilitated by the attachment of binding proteins to methylation sites and, subsequently, the association of the binding proteins with histone deacetylases. This interaction leads to chromatin remodeling and, ultimately, decreases access to transcription binding sites [10]. In nature, well-regulated DNA methylation is responsible for normal genetic processes such as the production of Barr bodies (inactivated X chromosomes), selective silencing of imprinted genes, cell differentiation and, by extension, tissue differentiation [11].

Strong associations between aberrant DNA methylation and various disorders such as neuropathies, malignancies, and metabolic dysfunctions such as obesity are reported [12]. Chief among the genes associated with the development of obesity are the leptin and adiponectin genes. Leptin is a protein hormone synthesized and secreted from adipose tissue that helps regulate body weight by reducing appetite [12]. To accomplish this, leptin stimulates leptin receptors on pro-opiomelanocortin (POMC) neurons in the hypothalamus [13] which then releases melanocyte-stimulating hormone (MSH) [14]. MSH acts on hypothalamic neurons expressing melanocortin-4 receptor (MC4R) thereby suppressing appetite via downstream signaling [15]. While many studies avoid linking the methylation status of the leptin gene to obesity in a cause-and-effect context, it is worth noting that a correlation has been made between the observation of hypomethylation of the leptin gene and the obesity phenotype [16-18]. As gene hypomethylation is typically linked to increased gene activity (decreased gene silencing), the relationship between leptin gene hypomethylation and obesity may reflect the leptin resistance observed in obese populations as dysregulated exposure to a stimulus can result in a decreased sensitivity of receptors to that stimulus [13]. In other words, being obese may cause increased leptin release as the body attempts to correct the excessive energy intake by upregulating appetite suppression to control tendencies to overindulge. When excessive consumption of energy substrates (food) persists, leptin levels may remain abnormally high as the body continues to try to curb eating. As a result, hypothalamic neurons may become desensitized to baseline levels of the hormone and manifest leptin resistance. Alternatively, it is possible that, through mechanisms requiring more research, hypothalamic neurons become resistant to leptin before the development of obesity. The resultant failure of appetite suppression could then lead to overeating and obesity. Knowing the role of leptin in appetite regulation makes this hormone an excellent candidate for continued research as targeting the activity of this gene may allow physicians to apply epigenetic principles to regulate the appetites of their obese patients.

In contrast to the *POMC* gene, activation of the neuropeptide Y (*NPY*) gene is associated with hunger and, therefore, increased food intake. In obese individuals, hypermethylation of *POMC* has been observed whereas *NPY* tends to be hypomethylated [17]. Since DNA methylation is most associated with gene silencing, it stands to reason that a cause-and-effect relationship potentially exists between obesity and excessive silencing of the satiety gene and/or insufficient silencing of the hunger gene. These findings further support the existence of a relationship between the obesity phenotype and the methylation status of genes that control the body’s desire for food and, therefore, food intake. *POMC* and *NPY* could, therefore, be used as pharmaceutical targets for reversing obesity. Drugs tailored towards the methylation status of *POMC* and/or *NPY* may be able to suppress a patient’s appetite and jumpstart weight loss simply by way of reducing food consumption.

Aberrant methylation of other genes has also been linked to obesity, such as the beta-3 adrenoceptor gene (*ADRB3*) [16] and the insulin-like growth factor-2 gene (*ILGF2*) [18]. *ADRB3* codes for receptors bearing the same name. In white adipose tissue, they are involved in the regulation of lipolysis while in brown adipose tissue they are involved in the regulation of thermogenesis. Studies conducted in *ADRB3*-knockout mice concluded that diminished activity of *ADRB3* led to abnormal lipolysis, increased lipid storage and obesity in the mice. In humans, ADRB3 deficiency has been associated with an increased risk of developing cardiovascular diseases and obesity [19]. Therefore, like *POMC* and *NPY*, altering the methylation status of* ADRB3* by therapeutic agents is another excellent candidate for continued research in the efforts against obesity. *ILGF2* plays a vital role in the regulation of body structure and tissue growth. The gene product, ILGF2, is a protein that activates insulin-like growth factor 1 (ILGF1) receptors. The activated ILGF1 receptors go on to promote anabolic processes within the body, such as skeletal muscle growth. The methylation status of ILGF2 is reported to correlate negatively with body mass index [20]. In other words, hypomethylation of *ILGF2* and, therefore, increased activation of ILGF1 receptors is associated with a higher BMI, which is consistent with fat storage as an anabolic process [20]. This information also places ILGF2, along with the ILGF1 receptor on the growing list of potential therapeutic targets against obesity.

Serotonin (5-HT) is a neurotransmitter produced by cells of both the central and peripheral nervous system that plays an important role in mood regulation, anxiety, emotional behavior, appetite, and metabolism [21]. SLC6A4 is a transport protein found on the membranes of presynaptic neurons and it is responsible for reuptake of serotonin from the synaptic cleft. In doing so, SLC6A4 regulates the concentration of serotonin in the synapse and, therefore, the strength and duration of serotonin signaling [22]. In mice, overexpression of SLC6A4 produced leaner animals. Meanwhile, knockout of the *SLC6A4* gene appears to result in the development of obesity, fatty liver disease, and insulin resistance [21]. Increased methylation of the *SLC6A4* promotor region is shown to be linked to the development of obesity in human subjects [22]. These findings are consistent with a known function of serotonin as a mediator of satiety and suggest that decreased methylation of *SLC6A4* should have the potential to reduce appetite and decrease the risk of overeating.

Hypoxia-inducible factor

Hypoxia-inducible factor (HIF) is a transcription factor triggered by decreased blood and/or tissue oxygen levels. HIF regulates many different intracellular and physiological processes by regulating the expression of a variety of genes. HIF is a protein complex composed of one of three alpha subunits (HIF1A, HIF3A or EPAS1) and a beta subunit (ARNT). While the main function of HIF is as described above, evidence continues to emerge in support of HIF’s role in energy balance, metabolic processes, and obesity. In genetically modified mice exposed to a high-fat diet, interruption of adipocyte HIF1A or ARNT, specifically, has been associated with decreased adipogenesis, prevention of obesity, and decreased instances of insulin resistance [23]. Therefore, HIF1A and ARNT may be reasonable targets in terms of preventative interventions in individuals with a genetic predisposition towards obesity. A connection between the methylation status of HIF3A and BMI has been demonstrated where the amount of HIF3A gene methylation exhibited a linear relationship to BMI [23]. It is thought that upregulation of *HIF3A* may be the result of the hypoxia associated with sleep apnea or other disordered breathing secondary to high BMI and, therefore, obesity [23]. A novel use of HIF3A may be as a biomarker for the likelihood of developing obesity or as a biomarker for diagnostic confirmation of obesity-induced sleep apnea. Notably, whereas this line of research did not define a cause-effect relationship between BMI and HIF3A, such a relationship may exist. In the event that increased methylation of *HIF3A* is found to be a cause of increased adiposity, then HIF3A may be considered a potential therapeutic target against the development of obesity.

Histone modification

Histones are basic globular proteins that are integral to DNA structure. For DNA to fit within cell nuclei, it must wrap itself around histone complexes. These DNA-histone complexes, known as nucleosomes, condense DNA into compact structures known as chromatin. Of the post-translational modifications associated with histones, acetylation and methylation are the most widely discussed within the context of the relationship between epigenetics and obesity. Histone acetylation results in upregulated gene expression as the DNA structure becomes more relaxed and, therefore, DNA is more accessible for transcription. In contrast, histone methylation may upregulate or downregulate gene expression by disrupting or enhancing the DNA-histone interaction [17]. Interestingly, it is the location and the extent of the histone methylation that determines the result of this post-translational modification. Enzyme families responsible for histone modifications include histone-specific acetyltransferases, deacetylases, methyltransferases and demethylases [17].

In one example of histone modification, liver cells extracted from animals fed a high-fat diet exhibited an increase in histone acetylation at the location of histone 3 lysine 9 (H3K9) and histone 3 lysine 18 (H3K18) in relation to the tumor necrosis factor (*TNF*) gene [17]. In other words, a high-fat diet has been associated with increased expression of TNF. One of the TNF proteins, more specifically TNF-a, is a cytokine produced by several different cell types such as macrophages and adipocytes. Studies have determined that increased TNF expression can be linked to obesity by its tendency to function as a mediator of insulin resistance [24]. Insulin resistance is a state in which the cells of the body do not exhibit their usual sensitivity to insulin and, as a result, do not uptake glucose from the blood as efficiently as they would otherwise. This leads to hyperinsulinemia (excessive insulin secretion) and can exacerbate or precipitate hyperglycemia [25]. Evidence supports the concept that, even in the presence of insulin resistance, adipocytes tend to maintain their sensitivity to insulin, unlike muscle and liver cells. As muscle and liver cells fail to absorb glucose from the blood, blood glucose levels remain abnormally high and insulin levels may rise in an attempt to correct the hyperglycemia. Adipocytes respond to the increased hormonal stimulation by increasing their glucose uptake and by the formation of new adipocytes to accommodate the excess energy available for storage [26]. In this way, histone acetylation with respect to *TNF *and the resultant insulin resistance may encourage the emergence of the obesity phenotype. Interestingly, caloric restriction is reported to revert the increase in acetylation to the normal type [17]. This finding lends further support to the notion that the modification of TNF is epigenetically regulated. It has also been hypothesized that insulin may function as a negative feedback hormone within the central nervous system (CNS) with respect to food intake [27]. It is thought that CNS insulin resistance may disrupt this feedback inhibition loop and result in an excess of food intake (total calories) and excessive fat storage. With this knowledge, healthcare workers may consider increasing community education on the association between a chronic high-fat diet, insulin resistance, and obesity especially as it pertains to diabetic patients. As the research suggests, focusing too heavily on controlling only carbohydrate intake would be to provide incomplete information to patients on how to truly avoid and/or reverse insulin resistance and obesity.

Like DNA methylation, histone modifications have also been associated with regulation of *POMC* and *NPY*. Decreased acetylation at the location of H3K9 in relation to *POMC* has been associated with obesity secondary to a high-fat diet [17]. As discussed previously, *POMC* is normally involved in appetite suppression through downstream signaling. Decreased acetylation of *POMC* could, therefore, lead to decreased expression of POMC, decreased appetite suppression and, subsequently, increased food intake. Enhanced acetylation at the H3K9 locus, correlated with NPY expression, underscores the importance of epigenetic mechanisms, wherein dietary inputs, particularly from a high-fat diet, can modulate gene expression patterns, thereby contributing to the development of obesity through intricate molecular pathways [17]. Since *NPY* is associated with hunger, increased acetylation of H3K9 could lead to increased production of the NPY peptide which would, in turn, increase hunger and food intake.

Thyroid hormone signaling

A connection between thyroid function and obesity has long been made. For example, it is well-documented that patients with hypothyroidism commonly experience weight gain. A common complaint of persons with confirmed hypothyroidism and on strict calorie-restricted diets is that they hardly eat and still gain weight. There are several contributing factors to weight gain experienced by hypothyroid patients, such as increased glycosaminoglycan deposition and the resultant increase in water retention in tissues. Importantly, as thyroid hormone has been confirmed as a critical supporter of energy expenditure and metabolism, it is not surprising that an overactive thyroid has been linked to weight loss while an underactive thyroid has been linked to weight gain [28].

The thyroid gland secretes both thyroxine (T4) and triiodothyronine (T3). Though T4 is secreted from the thyroid in larger amounts than T3, T4 is peripherally converted into T3 as needed and it is T3 that is functionally active. Early studies focused on the connection between food intake and thyroid hormone assert that T3 production was drastically increased in lean participants during periods of excessive food intake. Conversely, T3 was found to be decreased in periods of caloric deficit for lean and obese persons [28]. As thyroid hormone has been linked to energy expenditure, it follows that T3 would increase during overfeeding to encourage increased energy expenditure as a means of compensating for the increased energy intake [28]. The inverse of this logic is therefore applicable in the instance of caloric deficit. Further to this, animal studies have proposed a direct link between thyroid hormone and appetite regulation. When T3 was increased in the peripheral tissues it had a catabolic effect resulting in weight loss. On the other hand, central increase of T3 (administration of T3 into the hypothalamus) resulted in anabolic effects such as increased appetite leading to increased food intake and weight gain [28].

While knowledge concerning the function and effects of thyroid hormone appears to be vast and continuously expanding, much less appears to be known about the function and effects of thyroid hormone in terms of body composition from an epigenetic perspective. As previously discussed, POMC neurons regulate appetite through the release of MSH which binds MC4R receptors on target neurons. Although MC3R is another hypothalamic MSH receptor, MC4R has been most closely associated with regulation of body weight. Just as thyroid stimulating hormone influences the expression of thyroid hormone, thyroid hormone receptor (TR) association with thyroid receptor binding elements (TRE) has been found to modulate expression of MC4R in mice. By interacting with TREs found within the *MC4R* gene promoter region, T3-TR complexes have been observed to play a role in T3 suppression of MC4R protein expression [29].

In studies on the regulation of MC4R expression by thyroid hormone, offspring of mice fed high-fat diets had greater concentrations of circulating T3 [29], which is in accordance with our earlier discussion of T3. Further, BMI is shown to be correlated with decreased MC4R expression, increased T3 levels, and increased association of T3-TRb complexes with TREs at histone 3 lysine 27 (H3K27) in the *MC4R* promoter region. Histone acetylation is thought to cause exposure of the promoter region of *MCR4*, thereby permitting repressor molecules to bind to this site and decrease MC4R expression in the offspring of high-fat diet fed mice [29]. In this way, TRb acts as a transcription factor that regulates the expression of MC4R. Moreover, when T3 and T4 concentrations were pharmacologically lowered via administration of methimazole, a thyroid hormone biosynthesis inhibitor, body weight in high-fat diet exposed pups was reduced. Additionally, methimazole was found to prevent the downregulation of MC4R, the upregulation of T3-TRb complexing, and therefore, the acetylation of H3K27 [29]. These results demonstrate a potential epigenetic pathway to obesity which could be dependent on thyroid hormone. In the first instance, decreased expression of MC4R would lead to decreased MSH activity and, therefore, decreased satiety and increased likelihood of overeating. In the second instance, increased histone acetylation secondary to T3-TRb interactions with TREs would also decrease MC4R expression, thereby also decreasing MSH activity [29]. The administration of methimazole adds further support to the notion that thyroid hormone may be central to this mechanism by virtue of the finding that the drug created the opposite effects of T3. These findings suggest several potential therapeutic targets against obesity, mostly notably T3, H3K27 and MC4R, as manipulation of these molecules can be used to increase satiety, decrease food intake and prevent or reverse obesity.

Long non-coding RNAs

Non-coding RNAs are segments of genetic material made up of less than 200 nucleotides which are not translated into proteins but are still functionally relevant. Within the context of epigenetic influences on obesity, long non-coding RNAs (lncRNAs) and micro RNAs (miRNAs) are considered among the most relevant non-coding RNAs studied [17]. Long non-coding RNA molecules have been implicated in obesity insofar as their regulatory function in the metabolism of lipids, glucose, and cholesterol. Epigenetic factors affecting the function or bioavailability of lncRNAs and miRNAs can therefore have an impact on metabolism and either contribute to the development of obesity or act as biomarkers or therapeutic targets for obesity.

Lipid metabolism is essential for supporting the normal functioning of many biological processes because lipid metabolism generates essential energy metabolites. Aberrant lipid function, therefore, can not only lead to fat deposition but also a disruption of other metabolic processes. Long non-coding RNAs can influence lipid metabolism through their interactions with RNA, DNA and protein molecules [30]. Such lncRNAs may affect lipid metabolism by interacting with transcription factors and, as a result, altering their interaction with their target genes. In this manner, two lncRNAs, AK133540 and AK142386, have been proposed to be associated with the regulation of homeobox A3 (HOXA3) and acyl-CoA dehydrogenase 10 (ACAD10), respectively. While further research is necessary to elucidate the extent and mechanism of action of these long noncoding RNAs on HOXA3 and ACAD10, it is important to note that products of these two genes have been implicated in adipogenesis and energy metabolism. Although the precise mechanisms require further investigation, AK133540 and AK142386 could potentially modulate the expression of key genes, thereby influencing the differentiation of preadipocytes into adipocytes and/or the regulation of obesity-related metabolic pathways [31]. Given their implicated roles in lipogenesis, the lncRNAs AK133540 and AK142386 may emerge as promising targets in gene therapy for obesity, wherein modulating their expression or function could potentially alter adipogenic pathways and lipid storage. This would be a novel approach to manage and possibly mitigate the metabolic and morphological alterations associated with obesity.

In cholesterol metabolism, lncRNAs and miRNAs have been found to exhibit cross-talk that affects cholesterol homeostasis. For example, it has been determined that microRNA 140 (miR-140) increases the expression of a lncRNA known as NEAT1 [30]. miR-140’s upregulation of NEAT1 is reported to be critical for adipogenesis [32]. This information may prove to be clinically relevant as increased levels of NEAT1 or miR-140 may prove to be useful biomarkers for the detection of aberrant adipogenesis. Additionally, disruption of the signaling pathway between NEAT1 and miR-140 may provide yet another therapeutic avenue in relation to obesity. In view of the fundamental role of glucose in metabolic processes, dysfunctional glucose metabolism may lead to the development of obesity. The lncRNA betalinc1 (beta long intergenic noncoding RNA1) regulates transcription factors associated with pancreatic islet cells and, as a result, influence the differentiation of islet cells and insulin production [30]. Epigenetic modification of lncRNA betalinc1 may, therefore, make a person susceptible to either hypo- or hyper-insulinemia, hyperglycemia, and insulin resistance which can lead to obesity. A novel correlation between obesity and dysfunctional glucose homeostasis is reported to result from the downregulation of a long non-coding RNA 1810019D21Rik given the name RIOT (regulator of insulin transcription), a lncRNA found in pancreatic beta cells [33]. RIOT downregulation appears to inhibit DNA methyltransferase 3a degradation leading to increased methylation of the promoter region of Nkx6.1 [33]. Nkx6.1 is a transcription factor involved in the differentiation, development, and function of pancreatic beta cells [34]. Decreased insulin transcription and dysfunctional glucose regulation seems to be the consequence of increased methylation of the Nkx6.1 promoter [33]. Based on the foregoing, lncRNA RIOT and transcription factor Nkx6.1 can both be considered candidates for the management and/or reversal of obesity-induced diabetes.

Dietary factors

There are decades worth of research and discussion available on the implications of diet on physical and emotional health. It is common knowledge that a diet consisting of excessive calories, usually consistent with low fruit and vegetable content, is an almost certain antecedent to obesity. Less commonly known are the epigenetic modifications that follow obesity, which can lead to or increase the risk of other disorders and diseases. For example, consumption of a high-fat diet for five days is shown to increase DNA methylation of the transcription factor PPAR-gamma [17]. PPAR-gamma plays an essential role in optimal adipocyte performance, and decreased PPAR-gamma function has been associated with lipodystrophy, insulin resistance, and obesity [35]. Long-term exposure to a high-fat diet is also reported to upregulate histone deacetylase 9, leading to the disruption of adipocyte differentiation which may affect adipocytes by decreasing adiponectin synthesis [17]. Adipocyte adiponectin has several functions including support of insulin signaling, increased insulin sensitivity, and decreasing triglyceride levels in muscle tissue [36]. There is evidence in support of adiponectin having strong anti-atherosclerotic effects by inhibiting adhesion molecule expression, nuclear factor kappaB activation and macrophage scavenger receptor A-1 expression [36]. These findings suggest that epigenetic influences on both adiponectin and PPAR-gamma may predispose an individual to dysregulation of lipid metabolism, insulin resistance and obesity. 

Studies on the effects of diet on the development of cervical intraepithelial neoplasia (CIN) demonstrate hypermethylation of long interspersed nucleotide elements (LINE1s) of mononuclear cells found in peripheral blood linked to a significantly lower risk of being diagnosed with CIN. It is thought that unhealthy eating patterns, most notably those consistent with a Western diet, placed individuals at a higher risk of developing CIN as such dietary patterns are consistent with hypomethylation of LINE1 [37]. 

Just as substances of abuse cause a neuroadaptive response in the reward center of the brain, so too can enticing foods. The connection between the consumption of appetizing foods and the resultant pleasure experienced is known to be mediated by a sudden spike in dopamine (DA) levels within the mesolimbic system. The amount of dopamine released is highly correlated to the amount of gratification experienced. Impaired dopamine regulation can, therefore, lead to overeating even in the face of deleterious consequences - behaviors commonly observed in individuals with the obesity phenotype. Studies have shown the promoter region of dopamine transporter SLC6A3 to be hypermethylated after exposure to high-fat, high-carbohydrate diets. SLC6A3, a presynaptic membrane protein, is responsible for rapid reuptake of dopamine into presynaptic neurons, thereby limiting the action of DA [38]. Theoretically, methylation of the SLC6A3 gene, and therefore a decrease in the SLC6A3 membrane protein, is expected to prolong the activity of DA in the synapse. This might increase the pleasure and reward derived from the behavior of consuming a high-fat, high-sugar diet and positively reinforce the behavior. Unsurprisingly, such behaviors may encourage weight gain and lead to obesity.

Maternal factors

Evidence is emerging which supports the concept that the intrauterine environment exerts a significant influence on fetal development. The period of plasticity early on in the gestational period could prove beneficial for the developing offspring, as changes in the gestational environment that reflect the external environment would allow for preemptive adaptation to external stimuli. However, this susceptibility to adaptation may also be deleterious. The Developmental Origins of Health and Disease (DOHAD) hypothesis suggests that being exposed to a harmful environment while in the womb or during the early stages of life can have negative effects on one's health well into adulthood [18]. Subjugation to maternal obesity, famine during gestation, nutritional supplements, drugs, alcohol, or other chemical agents can trigger epigenetic alterations that impact the development of the embryo and placenta. Further, these epigenetic changes may alter biological processes that regulate adiposity. Based on current knowledge, the two main targets for epigenetic changes that influence fetal predisposition to obesity are regulating factors of adipocytes and regulating factors of food intake [39].

Like dopamine, endogenous opioids also participate in the sensations of gratification and pleasure generated in the mesolimbic system. While much is left to be discovered about the connection between the maternal environment and modified expression of neurotransmitters in offspring, emerging research lends support that epigenetic alterations may adversely affect endogenous opioid expression in offspring of mothers exposed to a high-fat diet. Studies suggest that maternal ingestion of appetizing foods during the gestational period, namely those foods high in fat, salt, and sugar, may promote a preference for such foods in the offspring [40]. It has been hypothesized that maternal exposure to high-fat foods may alter the expression of endogenous opioids in the mesolimbic system of offspring. This is evidenced by opioid receptor and pre-proenkephalin (PENK) expression changes in the offspring of high-fat fed mothers. Notably, the promoter region of *PENK* displays hypomethylation in offspring subjected to such maternal conditions. In accordance with our earlier discussion surrounding DNA methylation, hypomethylation of the *PENK* promoter corresponds with increased proenkephalin [40]. As research suggests that opioid receptor stimulants tend to increase the appetite and increase food intake [40], increased PENK in offspring could easily encourage overeating. Coupled with a preference for highly palatable foods, it is clear that maternal influences may support the development of obesity in offspring - a condition that may easily persist into adulthood.

Fetal nutrition is shown to exert influence over the activation of adipogenesis and lipogenesis. Excessive maternal nutrition may lead to a dramatic increase in PPAR-gamma, a transcription factor affecting the development of subcutaneous fat. PPAR-gamma activation has been positively and directly correlated with fetal blood glucose concentration [41]. Consequently, PPAR-gamma increased the expression of fetal lipoprotein lipase (LPL) and glyceraldehyde 3-phosphate dehydrogenase (G3PDH) which, in turn, promote incorporation of free fatty acids and glucose into adipocytes [41]. In a separate study, maternal nutrition was found to play a role in the generation of signaling molecules that affect NPY and POMC [39]. Similarly, offspring of pregnant mice exposed to a high-fat diet for four weeks showed increased methylation of the insulin receptor substrate 2 gene, which negatively impacts the normal functioning of insulin downstream. Additionally, hypomethylation of the mitogen-activated protein kinase 4 (*MAPK4*) gene was found [39]. These changes in methylation patterns were positively correlated with increased body weight, hyperglycemia, and insulin resistance in offspring [17]. Offspring of mothers fed a high-fat diet were found to have hyperinsulinemia, hyperglycemia, and increased LPL and leptin gene expressions [39]. Further support for an important influence of maternal diet on offspring phenotype comes in the form of babies born to women with gestational diabetes mellitus. Infants born to diabetic mothers are reported to possess significantly more adipose tissue and larger skinfold measurements compared to those exposed to undernutrition during the gestational period [39].

The importance of maternal health during the gestational period has always been emphasized for the well-being of the mother and for mitigating the risks involved in genetic disorders and diseases that have a clearly established cause-effect, such as alcohol fetal syndrome. However, healthcare providers may also consider equally emphasizing the importance of maternal health in terms of avoiding more insidious outcomes of an unfavorable maternal environment, such as fetal and childhood obesity that can persist into adult life. By making positive changes at the maternal level over the course of generations, it may be possible that the prevalence of obesity, especially in children, can be greatly reduced.

Physical activity

Physical activity has also been linked to epigenetic changes which support the development of obesity. Excessive and prolonged inactivity have been reported to decrease insulin sensitivity as well as promote dysfunctional energy metabolism and mitochondrial function. Decreased DNA methylation in the regulatory region of the PPAR-gamma coactivator 1-apha (*PGC1α*) gene is shown to be associated with upregulated expression of the PGC1α [17]. The PGC1α protein interacts with transcription factors to increase the transcription of PPAR-gamma [42]. As the PPAR-gamma protein is involved in lipid and glucose metabolism, it makes sense that exercise would induce the activity of this protein and that exercising would put the body in an energy deficit. PGC1α could, therefore, be a novel pharmaceutical target in obese patients who, based on the above, would likely have increased methylation of this coactivator. 

Skeletal muscle biopsies from obese and diabetic subjects before and after 16 weeks of aerobic or resistance exercise are reported to show decreased DNA methylation of the nuclear respiratory factor 1 (*NRF1*) gene, a transcription factor involved in the normal functioning of several metabolic genes [17]. The fatty acid synthase (FASN) gene appears to exhibit increased methylation which may explain why circulating lipids were reduced and metabolic function was improved. Resistance training resulted in the modification of microRNAs involved in metabolism and methylation of the solute carrier family 2 (*SLCA2*) gene involved in mitochondrial function and metabolism of fatty acids, which was found to be increased. These epigenetic changes were associated with improved glucose utilization and decreased lipid content of the skeletal muscles [17]. 

Table 1 summarizes the target genes and epigenetic modifications purported to reflect the important environment-gene interactions leading to obesity.

**Table 1:** Target genes, epigenetic modifications and functional outcomes. POMC=pro-opiomelanocortin; NPY=neuropeptide Y; ADRB3=Beta-3 adenoreceptor; H3K9=histone-3 lysine-9; ILGF2=insulin-like growth factor2; SLC6A4=solute carrier family serotonin transporter6A4; HIF3A=hypoxia-inducible factor3A; TNF=tumor necrosis factor; H3K18=histone-3 lysine-18; MC4R=melanocortin-4 receptor; T3-TRb=triiodothyronine-thyroid receptor-b; TRE=thyroid receptor binding element; H3K27=histone-3 lysine-27; HOXA3=homeobox A3; ACAD10=acyl-CoA dehydrogenase-10; lncRNA=long noncoding RNA; NEAT1=nuclear paraspeckle assembly transcript-1; mIR-140=micro RNA-140; lncRNA betalinc1=beta long intergenic noncoding RNA1; RIOT=regulator of insulin transcription; PPAR-gamma=peroxisome proliferator activated receptor-gamma; LPL=lipoportein lipase; G3PDH=glyceraldehyde 3-phosphate dehydrogenase; PGC1α=PPAR-gamma coactivator 1-alpha; SLC6A3=solute carrier family-3; PENK=pre-proenkephalin; MAPK4=mitogen activated protein kinase-4; NRF1=nuclear respiratory factor-1; FASN=fatty acid synthase

Target gene	Epigenetic modification	Functional outcomes
POMC	Hypermethylated	Inhibits satiety
	Decreased acetylation of H3K9 related to POMC	Increased food intake
NPY	Hypomethylated	Increases hunger and food intake
	Increased acetylation of H3K9 related to NPY	
ADRB3	Reduced activity of ADRB3	Abnormal lipolysis, increased lipid storage, and obesity.
ILGF2	Hypomethylation of ILGF2	Increased BMI
SLC6A4	Increased methylation of the SLC6A4 promotor region	Obesity
HIF3A	Increased methylation	Obesity associated with breathing disorders
TNF	Increased histone acetylation at H3K9 and H3K18 leads to increased TNF expression	Mediates insulin resistance
MC4R	Increased association of T3-TRb complexes with TREs at H3K27 in the MC4R promoter region reduces the MC4R expression and leads to reduced MSH activity	Overeating
HOXA3 and ACAD10	Two lncRNAs, AK133540 and AK142386 modulate the interactions of transcription factors with their target genes	Lipogenesis
NEAT1 or miR-140	miR-140 upregulates the expression of NEAT1	Adipogenesis
lncRNA betalinc1	Regulates transcription factors associated with pancreatic islet cells	Alters glucose metabolism leading to obesity
RIOT Nkx6.1	Downregulation of RIOT results in increased methylation of the promoter region of Nkx6.1, a transcription factor crucial for the differentiation, development, and function of pancreatic beta cells	Obesity-induced diabetes
PPAR-gamma	Increased DNA methylation of the transcription factor PPAR-gamma	Dysregulated lipid metabolism, insulin resistance, and obesity
	PPAR-gamma activation is associated with increased expression of fetal LPL and G3PDH	Activation of adipogenesis and lipogenesis influencing the development of subcutaneous fat
	PGC1α interacts with transcription factors to enhance the transcription of PPAR-gamma	Dysregulates lipid and glucose metabolism
SLC6A3	Hypermethylation of the SLC6A3 gene decreases the SLC6A3 membrane protein, causing prolonged dopamine activity	Overeating even when aware of the negative consequences
PENK	Hypomethylation of the PENK promoter corresponding to increased proenkephalin	Causing overeating in offspring
MAPK4	Hypomethylation of MAPK4 gene	Increased body weight, hyperglycemia, and insulin resistance in offspring
NRF1	Decreased DNA methylation of the NRF1 gene, a transcription factor	Affects various metabolic genes
FASN	Increased methylation of the FASN gene	Reduces circulating lipids and the improves metabolic function

Body weight set point

The concept of a body weight "set point" postulates that the body has an innate mechanism that regulates weight within a specific range. Evidence is emerging that epigenetic processes could play a pivotal role in determining this set point. The body weight set point theory argues that our bodies are designed to maintain a specific weight range, and any deviations from this range activate compensatory mechanisms to restore weight to its set point [43]. Epigenetics has emerged as a key regulator of various physiological processes including body weight regulation [44]. Findings mentioned earlier in this review suggest that altered DNA methylation patterns can shift the body's weight set point by influencing appetite control by virtue of DNA methylation influencing the expression of genes related to appetite and energy balance. For example, methylation of the promoter region of POMC, which encodes a precursor molecule of the appetite-suppressing hormone alpha-MSH, is associated with increased BMI [45]. In reviewing the roles of histone modifications linked to genes governing energy expenditure, it seems likely that activity of histones which regulate transcription of genes related to metabolism may influence the body weight set point [46]. Non-coding RNAs, particularly miRNAs, have been implicated in adipocyte differentiation (adipogenesis). For instance, miR-143 promotes adipocyte differentiation and is upregulated in the adipose tissue of obese mice [47]. By influencing adipogenesis, miRNAs can potentially affect the amount of adipose tissue and thereby regulate body weight set point. Early-life nutrition can induce long-term epigenetic changes influencing the predisposition to obesity. Studies have shown that malnutrition during fetal development in humans exposed to famine can lead to epigenetic modifications, affecting genes related to metabolism and appetite, which might establish a higher body weight set point in adulthood [48]. Taken together, these findings suggest that epigenetic mechanisms, encompassing DNA methylation, histone modifications, and non-coding RNAs, present compelling evidence for various roles in regulating the body weight set point. Understanding these mechanisms could open avenues for targeted interventions to treat obesity and related metabolic disorders.

There appear to be several pathways for epigenetic programming of the adult body weight set point. Alterations in the fatty acid composition of breast milk, differences in the metabolic and hormonal profiles and changes in the gut microbiota of neonatal animals and humans may all play roles in epigenetic programming of the set point [49]. Breast milk lipids, especially fatty acids, are vital for infant development, meeting about half of their energy needs. The polyunsaturated fatty acids (PUFAs) in breast milk can, no doubt, impact an infant's fat deposition and growth. Over the past 30 years, there's been an increased omega-6/omega-3 PUFA ratio in human breast milk, correlating with heightened maternal consumption of refined vegetable oils rich in omega-6 PUFAs [50]. This connection emphasizes the obesogenic effect of omega-6 PUFAs during the perinatal period. Fatty acids can also influence gene expression through lipid-sensing transcription factors and resultant DNA methylome changes. The PPAR family, especially PPARα, is shown to play an important role in these epigenetic alterations. PPARα, when activated, is shown to trigger DNA demethylation of its target genes, like the *Fgf21* gene, which influences energy homeostasis [51]. Studies in mice have revealed the importance of PPARα in attenuating obesity caused by high-fat diets [52]. Indeed, changes in breast milk fatty acid composition associated with maternal high-fat feeding have been linked to DNA demethylation in genes expressed specifically in white adipose tissue [53]. PPARγ also influences DNA demethylation. In adipocytes taken from obese mice, downregulation of the neutral amino acid transporter SLC1A5 and reductions in adipocyte uptake of the amino acids glutamine and methionine were associated with dysfunction of Bmal1, a key circadian rhythm regulator [54]. Indeed, circadian rhythm disruption is recognized as a promoter of obesity in women [55], as well as in resident physicians of both genders and male firefighters exposed to shift work [56].

Recent findings highlight the role of human milk oligosaccharides (HMOs) in reducing infant adiposity, suggesting their potential in protecting against obesity when breastfeeding. Unlike infant formulas, HMOs are abundant in human milk and may function as prebiotics, potentially aiding in reducing fat build-up by fostering healthy gut microbiota and their resultant metabolites like short-chain fatty acids (SCFAs). SCFAs like butyric and formic acid in breast milk, are reported to inversely correlate with infant BMI [57]. The possible epigenetic impact of early postnatal nutrition could, therefore, stem from alterations in gut microbiota during this developmental phase. Future studies should address the intricate links between postnatal nutrition, gut microbiota, and their potential role in epigenetic programming of the adult body weight set point.

Postnatal programming of the adult body weight set point may also be mediated by epigenetic influences on insulin secretion. Hyperinsulinemia in the offspring of various animals has been linked to transference of breast milk hormones such as insulin and leptin [58]. A role for insulin signaling in the postnatal epigenetic reprogramming of the liver is suggested by profound effects of demethylation on insulin receptor deletion during liver maturation [59]. In that regard, a collection of insulin target genes are shown to be impacted in postnatal programming models, thereby providing evidence of the far-reaching consequences of hormonal alterations in neonates [60]. Gender-based metabolic programming is shown to result from interactions between testosterone, DNMTs and ten-eleven translocation (TET) enzymes in the ventromedial nucleus satiety center, among other brain areas [61]. This line of research may someday help explain gender-based differences in predilections for obesity in men compared to women. These findings on gender-based metabolic programming, involving interactions between testosterone, DNMTs, and TET enzymes within key brain regions open up new avenues for understanding not only obesity but also related health conditions, such as autoimmune diseases and post-maternity weight management, which exhibit gender-based disparities. For example, autoimmune diseases, including thyroid disorders like Hashimoto's and Graves' disease, have a higher prevalence in women compared to men. Gender-specific metabolic programming could potentially contribute to these differences. It is, therefore, plausible that the same molecular interactions mentioned in the study might influence the immune system's response, making women more susceptible to autoimmune diseases, including those affecting the thyroid gland. Another gender-specific aspect of metabolism relates to the post-maternity period. Women often face unique challenges in managing their weight after pregnancy. Hormonal changes, including fluctuations in estrogen and progesterone, could interact with the brain's metabolic programming mechanisms. This research may provide insights into why women tend to experience different post-pregnancy weight trajectories compared to men. Such knowledge could lead to more targeted interventions and therapies, ultimately improving the health outcomes of individuals, particularly women, who are disproportionately affected by these conditions. Additionally, a role for high blood glucose levels interacting with TET enzymes involved in DNA demethylation and O-GlcNAc transferase (OGT) involved in important post-translational modifications of proteins, makes a compelling argument for a direct influence of metabolic stimuli such as hyperinsulinemia and/or hyperglycemia on epigenetic modifications which could influence the adult body weight set point [62].

Table 2 summarizes the epigenetic modification that can influence the body weight set point and lead to obesity. 

**Table 2:** Summary of epigenetic factors that influence body weight set point. POMC=pro-opiomelanocortin; MSH=melanocyte stimulating hormone; MiR-143=micro RNA-143; PPARα=peroxisome proliferator-activated receptor alpha; SLC1A5=solute carrier family 1 member 5; DNMTs=DNA methyltransferases; TET=ten-eleven translocation; OGT=O-GlcNAc transferase; PUFA=polyunsaturated fatty acids

Factors	Mechanisms
Adult factors	
Appetite	DNA methylation of the promoter region of POMC which encodes an appetite suppressor gene alpha-MSH
Metabolism	Modification of histone regulating transcription of gene related to metabolism
Adipocytes	Non-coding RNAs, MiR-143 promotes adipocyte differentiation and impacts adipogenesis
High-fat diet	PPARα can initiate DNA methylation in the Fgf21 gene and increase obesity caused by high-fat diets
Circadian rhythm	Dysfunction of Bmal1, a crucial circadian rhythm regulator, was linked to downregulation of the neutral amino acid transporter SLC1A5 and reductions in uptake of the amino acids glutamine and methionine in adipocytes
Gender	Metabolic programming due to the interaction between testosterone, DNMTs, and TET enzymes in the ventromedial satiety center
Hyperglycemia and hyperinsulinemia	High blood glucose levels interacting with TET enzymes involved in DNA demethylation and OGT affecting post-translational modifications of proteins may directly influence the adult body weight set point
Neonatal factors	
Fetal malnutrition	Epigenetic modification of genes related to metabolism and appetite
Neonatal influences	Changes in the fatty acid composition of breast milk and changes in gut microbiota can cause epigenetic programming of set point
Breast milk	Increased omega-6/omega-3 PUFA ratio in breast milk due to increased consumption of refined vegetable oils causes DNA methylation in genes of white adipose tissue
Infant formula	Does not contain human milk oligosaccharides and short-chain fatty acids like butyric acid and formic acid
Hormonal alteration	Transfer of hormones like insulin and leptin through breast milk can cause postnatal epigenetic reprogramming of the liver, demethylation on insulin receptor deletion during liver maturation

Summary

**Figure 1:**
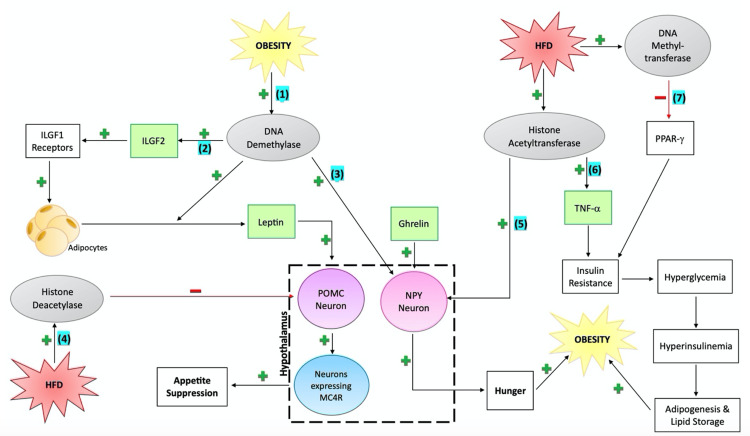
Seven epigenetic mechanisms of obesity. (1) Obesity induced hypomethylation of leptin gene leading to ­leptin release, increased stimulation of POMC neurons and MSH release, ­increased stimulation of neurons expressing MC4R, increased appetite suppression to decrease energy intake; (2) Demethylation of ILGF2 gene leading to increased ILGF2 levels, increased ILGF1 receptor stimulation and increased adipogenesis; (3) Hypomethylation of NPY genes leads to increased NPY release, increased hunger and a tendency to over-eat; (4) HFD leads to decreased acetylation of *POMC* resulting in decreased POMC release and decreased appetite suppression; (5) HFD leads to increased acetylation of *NPY* resulting in increased NPY release, increased hunger and a tendency to over-eat; (6) HFD leads to increased acetylation of TNF-alpha, increased TNFalpha expression, insulin resistance and increased adipogenesis and lipid storage; (7) HFD leads to increased methylation of *PPAR-gamma*, decreased PPAR-gamma expression, insulin resistance and increased adipogenesis and lipid storage. ILGF2=insulin like growth factor 2; ILGF1=insulin like growth factor 1; POMC=pro-opiomelanocortin; MSH=melanocytes stimulating hormone, MC4R=melanocortin-4 receptor; NPY=neuropeptide Y, HFD=high-fat diet; TNF-alpha=tumor necrosis factor alpha; PPAR-gamma=peroxisome proliferator-activated receptor gamma Image credit: Erin N.L. Smith

Figure 1 depicts the relationships between seven epigenetic mechanisms shown, in this review, to be involved in the development of obesity.

DNA methylation, histone modifications, and the influence of the prenatal environment are some of the mechanisms by which epigenetic changes occur. Regulated DNA methylation is necessary for a number of biological processes, such as cell differentiation. Conversely, dysregulated DNA methylation has been implicated in the pathogenesis of obesity because it may lead to leptin resistance through hypomethylation of *POMC*. Aberrant methylation of *NPY*, *ADRB3*, *IGF1*, and *HIF3a* have all been linked to obesity. Decreased histone acetylation of *POMC* and increased acetylation of *NPY *have been linked to obesity following exposure to a high-fat diet. Epigenetic evidence of intergenerational effects include hypomethylation of *POMC*, changes in expression of melanocortin and insulin receptors, as well as altered gene expression of appetite-regulating dopamine and opioid-related genes in the offspring of obese mothers or those exposed to a high-fat diet. lncRNAs are shown to be associated with disruptions in lipid, glucose and cholesterol metabolism and several lncRNAs are more highly expressed in obese individuals compared to their normal body weight counterparts. The most widely reported and discussed epigenetic factors involve diet and physical activity involving hypomethylation of *POMC*, *NPY*, *ADRB3*, *IGF*, *HIF3a* and an activator of *PPAR-gamma*.

## Conclusions

Obesity has reached epidemic proportions in the developed world and its incidence continues to rise on a yearly basis. Given that obesity is now classified as a disease, as well as a major risk factor for diabetes mellitus, cardiovascular disease, cancer, and cerebrovascular disease, it is imperative that we attempt to understand all facets of the pathogenesis of obesity in our quest to decrease its prevalence. The role of epigenetics in obesity has recently come to center stage and continues to be an area where further study would prove most beneficial in enhancing our understanding not only of how obesity occurs, but also of potential treatments and preventive interventions. While much has been uncovered thus far, continued investigations are necessary to establish true cause-effect relationships between epigenetic markers and the development of obesity. Such evidence of environment-gene interaction could lead to the discovery of novel therapeutic targets in the fight against obesity. Ongoing research to establish the cause-effect links between epigenetic markers and obesity holds immense promise, as it could unveil the critical role of environmental factors in influencing gene expression. This deeper understanding of environment-gene interactions may pave the way for the identification of innovative therapeutic strategies aimed at combating obesity by targeting these epigenetic mechanisms. Given the prevalence of obesity on a global scale it is vital to use novel methods and models to elucidate the epigenetic mechanisms affecting the development and progression of this disease.

## References

[1] Herrera BM, Keildson S, Lindgren CM (2011). Genetics and epigenetics of obesity. Maturitas.

[2] McAllister EJ, Dhurandhar NV, Keith SW (2009). Ten putative contributors to the obesity epidemic. Crit Rev Food Sci Nutr.

[3] (1998). NHLBI Obesity Education Initiative Expert Panel on the Identification, Evaluation, and Treatment of Obesity in Adults (US). Clinical guidelines for the identification, evaluation, and treatment of overweight and obesity in adults-the evidence report. Obes Res.

[4] Hales CM, Carroll MD, Fryar CD (2020). Prevalence of obesity and severe obesity among adults: United States, 2017-2018. NCHS Data Brief.

[5] Slomko H, Heo HJ, Einstein FH (2012). Minireview: epigenetics of obesity and diabetes in humans. Endocrinology.

[6] Murr R (2010). Interplay between different epigenetic modifications and mechanisms. Adv Genet.

[7] Vo AT, Millis RM (2012). Epigenetics and breast cancers. Obstet Gynecol Int.

[8] Meeran SM, Ahmed A, Tollefsbol TO (2010). Epigenetic targets of bioactive dietary components for cancer prevention and therapy. Clin Epigenetics.

[9] Newman M, Blyth BJ, Hussey DJ, Jardine D, Sykes PJ, Ormsby RJ (2012). Sensitive quantitative analysis of murine LINE1 DNA methylation using high resolution melt analysis. Epigenetics.

[10] Newell-Price J, Clark AJ, King P (2000). DNA methylation and silencing of gene expression. Trends Endocrinol Metab.

[11] de Mello VD, Pulkkinen L, Lalli M, Kolehmainen M, Pihlajamäki J, Uusitupa M (2014). DNA methylation in obesity and type 2 diabetes. Ann Med.

[12] Al-hussaniy H, Altalebi RR, Tylor FM (2022). Leptin hormone: in brief. Med Pharm J.

[13] Friedman JM (2019). Leptin and the endocrine control of energy balance. Nat Metab.

[14] Forbes S, Bui S, Robinson BR, Hochgeschwender U, Brennan MB (2001). Integrated control of appetite and fat metabolism by the leptin-proopiomelanocortin pathway. Proc Natl Acad Sci U S A.

[15] Sadashiv Sadashiv, Modi A, Khokhar M (2021). Leptin DNA methylation and its association with metabolic risk factors in a northwest Indian obese population. J Obes Metab Syndr.

[16] Samblas M, Milagro FI, Martínez A (2019). DNA methylation markers in obesity, metabolic syndrome, and weight loss. Epigenetics.

[17] Mahmoud AM (2022). An overview of epigenetics in obesity: the role of lifestyle and therapeutic interventions. Int J Mol Sci.

[18] Obri A, Serra D, Herrero L, Mera P (2020). The role of epigenetics in the development of obesity. Biochem Pharmacol.

[19] Guay SP, Brisson D, Lamarche B (2014). ADRB3 gene promoter DNA methylation in blood and visceral adipose tissue is associated with metabolic disturbances in men. Epigenomics.

[20] Ács O, Péterfia B, Hollósi P, Luczay A, Török D, Szabó A (2017). Methylation status of CYP27B1 and IGF2 correlate to BMI SDS in children with obesity. Obes Facts.

[21] Lillycrop KA, Garratt ES, Titcombe P (2019). Differential SLC6A4 methylation: a predictive epigenetic marker of adiposity from birth to adulthood. Int J Obes (Lond).

[22] Zhao J, Goldberg J, Vaccarino V (2013). Promoter methylation of serotonin transporter gene is associated with obesity measures: a monozygotic twin study. Int J Obes (Lond).

[23] Dick KJ, Nelson CP, Tsaprouni L (2014). DNA methylation and body-mass index: a genome-wide analysis. Lancet.

[24] Hotamisligil GS, Arner P, Caro JF, Atkinson RL, Spiegelman BM (1995). Increased adipose tissue expression of tumor necrosis factor-alpha in human obesity and insulin resistance. J Clin Invest.

[25] Freeman AM, Acevedo LA, Pennings N (2023). Insulin resistance. StatPearls [Internet].

[26] Isganaitis E, Lustig RH (2005). Fast food, central nervous system insulin resistance, and obesity. Arterioscler Thromb Vasc Biol.

[27] Schwartz MW, Boyko EJ, Kahn SE (1995). Reduced insulin secretion: an independent predictor of body weight gain. J Clin Endocrinol Metab.

[28] Santini F, Marzullo P, Rotondi M (2014). Mechanisms in endocrinology: the crosstalk between thyroid gland and adipose tissue: signal integration in health and disease. Eur J Endocrinol.

[29] Tabachnik T, Kisliouk T, Marco A, Meiri N, Weller A (2017). Thyroid hormone-dependent epigenetic regulation of melanocortin 4 receptor levels in female offspring of obese rats. Endocrinology.

[30] Lu Q, Guo P, Liu A (2021). The role of long noncoding RNA in lipid, cholesterol, and glucose metabolism and treatment of obesity syndrome. Med Res Rev.

[31] Chen J, Cui X, Shi C (2015). Differential lncRNA expression profiles in brown and white adipose tissues. Mol Genet Genomics.

[32] Gernapudi R, Wolfson B, Zhang Y, Yao Y, Yang P, Asahara H, Zhou Q (2016). MicroRNA 140 promotes expression of long noncoding RNA NEAT1 in adipogenesis. Mol Cell Biol.

[33] Zhang FF, Liu YH, Wang DW (2020). Obesity-induced reduced expression of the lncRNA ROIT impairs insulin transcription by downregulation of Nkx6.1 methylation. Diabetologia.

[34] Aigha II, Abdelalim EM (2020). NKX6.1 transcription factor: a crucial regulator of pancreatic β cell development, identity, and proliferation. Stem Cell Res Ther.

[35] Corrales P, Vidal-Puig A, Medina-Gómez G (2018). PPARs and metabolic disorders associated with challenged adipose tissue plasticity. Int J Mol Sci.

[36] Kadowaki T, Yamauchi T (2005). Adiponectin and adiponectin receptors. Endocr Rev.

[37] Piyathilake CJ, Badiga S, Kabagambe EK, Azuero A, Alvarez RD, Johanning GL, Partridge EE (2012). A dietary pattern associated with LINE-1 methylation alters the risk of developing cervical intraepithelial neoplasia. Cancer Prev Res (Phila).

[38] Ramos-Lopez O, Riezu-Boj JI, Milagro FI, Martinez JA (2018). Dopamine gene methylation patterns are associated with obesity markers and carbohydrate intake. Brain Behav.

[39] Campión J, Milagro F, Martínez JA (2010). Epigenetics and obesity. Prog Mol Biol Transl Sci.

[40] Vucetic Z, Kimmel J, Totoki K, Hollenbeck E, Reyes TM (2010). Maternal high-fat diet alters methylation and gene expression of dopamine and opioid-related genes. Endocrinology.

[41] Muhlhausler BS, Duffield JA, McMillen IC (2007). Increased maternal nutrition stimulates peroxisome proliferator activated receptor-gamma, adiponectin, and leptin messenger ribonucleic acid expression in adipose tissue before birth. Endocrinology.

[42] Liang H, Ward WF (2006). PGC-1alpha: a key regulator of energy metabolism. Adv Physiol Educ.

[43] Speakman JR, Levitsky DA, Allison DB (2011). Set points, settling points and some alternative models: theoretical options to understand how genes and environments combine to regulate body adiposity. Dis Model Mech.

[44] Feinberg AP (2007). Phenotypic plasticity and the epigenetics of human disease. Nature.

[45] Widiker S, Karst S, Wagener A, Brockmann GA (2010). High-fat diet leads to a decreased methylation of the Mc4r gene in the obese BFMI and the lean B6 mouse lines. J Appl Genet.

[46] McGee SL, Hargreaves M (2020). Exercise adaptations: molecular mechanisms and potential targets for therapeutic benefit. Nat Rev Endocrinol.

[47] Esau C, Kang X, Peralta E (2004). MicroRNA-143 regulates adipocyte differentiation. J Biol Chem.

[48] Heijmans BT, Tobi EW, Stein AD (2008). Persistent epigenetic differences associated with prenatal exposure to famine in humans. Proc Natl Acad Sci U S A.

[49] Marousez L, Lesage J, Eberlé D (2019). Epigenetics: linking early postnatal nutrition to obesity programming?. Nutrients.

[50] Simopoulos AP (2016). An increase in the omega-6/omega-3 fatty acid ratio increases the risk for obesity. Nutrients.

[51] Kim JH, Wahyudi LD, Kim KK, Gonzalez FJ (2016). PPARα activation drives demethylation of the CpG islands of the Gadd45b promoter in the mouse liver. Biochem Biophys Res Commun.

[52] Hinds TD Jr, Kipp ZA, Xu M (2021). Adipose-specific PPARα knockout mice have increased lipogenesis by PASK-SREBP1 signaling and a polarity shift to inflammatory macrophages in white adipose tissue. Cells.

[53] Butruille L, Marousez L, Pourpe C (2019). Maternal high-fat diet during suckling programs visceral adiposity and epigenetic regulation of adipose tissue stearoyl-CoA desaturase-1 in offspring. Int J Obes (Lond).

[54] Wang S, Lin Y, Gao L (2022). PPAR-γ integrates obesity and adipocyte clock through epigenetic regulation of Bmal1. Theranostics.

[55] Al-Safi ZA, Polotsky A, Chosich J, Roth L, Allshouse AA, Bradford AP, Santoro N (2018). Evidence for disruption of normal circadian cortisol rhythm in women with obesity. Gynecol Endocrinol.

[56] Mota MC, De-Souza DA, Rossato LT (2013). Dietary patterns, metabolic markers and subjective sleep measures in resident physicians. Chronobiol Int.

[57] Sundberg N, Millis RM (2023). A study of diurnal cortisol adaptations in sleep-deprived firefighters during a 72-hour work shift: a case series. Cureus.

[58] Gao Y, Davis B, Zhu W, Zheng N, Meng D, Walker WA (2021). Short-chain fatty acid butyrate, a breast milk metabolite, enhances immature intestinal barrier function genes in response to inflammation in vitro and in vivo. Am J Physiol Gastrointest Liver Physiol.

[59] Kahraman S, Dirice E, De Jesus DF, Hu J, Kulkarni RN (2014). Maternal insulin resistance and transient hyperglycemia impact the metabolic and endocrine phenotypes of offspring. Am J Physiol Endocrinol Metab.

[60] Reizel Y, Morgan A, Gao L (2021). FoxA-dependent demethylation of DNA initiates epigenetic memory of cellular identity. Dev Cell.

[61] Nigi L, Grieco GE, Ventriglia G (2018). microRNAs as regulators of insulin signaling: research updates and potential therapeutic perspectives in type 2 diabetes. Int J Mol Sci.

[62] Bauer C, Göbel K, Nagaraj N (2015). Phosphorylation of TET proteins is regulated via O-GlcNAcylation by the O-linked N-acetylglucosamine transferase (OGT). J Biol Chem.

